# Estimation of the ultimate tensile strength and yield strength for the pure metals and alloys by using the acoustic wave properties

**DOI:** 10.1038/s41598-020-69387-z

**Published:** 2020-07-29

**Authors:** Arshed Abdulhamed Mohammed, Sallehuddin Mohamed Haris, Wessam Al Azzawi

**Affiliations:** 1 Department of Materials Engineering College of Engineering, University of Diyala, Baquba, Diyala Iraq; 2 Department of Mechanical & Manufacturing Engineering, Faculty of Engineering & the Built Environment, University Kebangsaan Malaysia, 43600 UKM Bangi, Selangor Malaysia

**Keywords:** Engineering, Materials science

## Abstract

In this paper, the acoustic impedance property has been employed to predict the ultimate tensile strength (UTS) and yield strength (YS) of pure metals and alloys. Novel algorithms were developed, depending on three experimentally measured parameters, and programmed in a MATLAB code. The measured parameters are longitudinal wave velocity of the metal, density, and crystal structure. 19-samples were considered in the study and divided into 3-groups according to their crystal structure; 7-FCC, 6-BCC, and 6-HCB. X-ray diffraction was used to examine the crystal structure of each sample of each group, while longitudinal wave velocity and metals’ density were measured experimentally. A comparison between mechanical properties predicted by the model and the ASTM standards was done to investigate the validity of the model. Furthermore, predicted stress–strain curves were compared with corresponding curves in the pieces literature as an additional validation check. The results revealed the excellence of the model with 85–99% prediction accuracy. The study also proved that if metals are grouped according to their crystal structure, a relation between UTS, YS, and modulus of elasticity (E) properties and wave pressure transmission coefficient (Tr) could be formulated.

## Introduction

In recent years, many studies tried to estimate the yield stress (YS) and ultimate tensile strength (UTS) of metals and alloys without using tensile tests^1,2^. succeeded in test YS and UTS, for steel alloys only by using a small punch test. Also, Palkovic et al.^3^ evaluated YS and toughness for steel pipelines by using non-destructive evaluation. Kancaa et al.^4^ used genetic expression programming to predict YS and UTS for cold-rolled steel. All these studies and others remained limited in steel and its alloys. In another hand, many resources focused on the tensile resistance of materials and any other material properties related to this resistance. The tensile resistance of any material, such as the fabric or any other material at room temperature depends on two things. The first is the type of material made from, and the other is the shape of the spun of this material^5,6^. The same thing is for the pure metals, where the resistance of the metals depends on the crystal structure and some of the mechanical properties.

In fact, the crystal structure and grain boundary types play a decisive role in the distinct slip mode of crystalline materials^7^; therefore it has an essential role in micro deformation mode. Many studies such as^8,9^ focused on the effect of microstructure grain sizes on the flow of stress to explain the mechanism of deformation during the tensile test, therefore Tanga et al.^8^ used pure metals such as Fe, Cu, and Ti to illustration this mechanism. This study provides another point of view to study and draw the behavior of pure metals and alloys, where, it focused on crystal structure as a parameter of grain size and grain boundary types then related it with acoustic impedance ($Z=\rho \times {C}_{L}$), Ys and UTS.

On the other hand, the important properties in tensile tests are the modulus of elasticity (E) and density (ρ) of this metal^10^. Those two properties (E and ρ) were employed in many studies to find other material properties.

This study used acoustic waves to calculate E, YS, and UTS required to draw the stress–strain curve which is one of the aims of this study, where this study can serve in many applications of the non-destructive evaluation applications^11,12^ such as testing the strength of pipeline^3^ and boiler tubes^13^, which face difficulty in testing directly by using normal tensile tests. Many studies have tried to consider this technique (the acoustic wave’s tests) in recent years, though there was always a margin of miss-match between the predicted and actual values. Many studies have referred to this discrepancy and tried to justify it by naming the E value calculated from the tensile test as static modulus of elasticity (E_s_) and E calculated from acoustic wave’s techniques as dynamic modulus of elasticity (E_D_), where $\left({E}_{D}=\frac{{C}_{L}^{2}\rho (1+\nu )(1-2\nu )}{(1-\nu )}\right)$. Ciccotti and Mulargia^14^ compared between E_s_ for seismogenic rocks (in the Italian Apennines) with E_D_ for the same material and found a 10% difference between them. Further, Builes et al.^15^ reported that the difference between the E_s_ and E_D_ increases with the density of the specimens. In the same context, many other published papers focused on this difference^16–21^. Mohammed et al.^22^ reported that the refractory metals have body force, therefore the equation of E_D_ cannot be used in the calculation of the modulus of elasticity because this equation neglected the term of body forces in the origin of this equation, where the origin of this equation is Navier governing equation. It is worth mentioning that, many of the offered devices in the global markets that used acoustic waves techniques such as 38DL PLUS^23^, 58-E4800, MATEST (C372M)…etc., still use E_D_ equation in finding some of the mechanical properties such as E, ν and other several mechanical properties for solid industry materials. This because these devices give correct results for normal materials such as Al, Fe, Zn.etc., however, for metals such as refractory metals, the results are incorrect^22^. Mohammed et al.^24^ succeeded in the calculation of the E for many metals and alloys, through finding a relationship between E × ρ and the pressure transmitted coefficient. Even though of this succeeding in finding E however, Mohammed et al.^24^ did not mention anything about the estimation of the YS and UTS for these metals, despite its importance. This study avoided the problem of equation E_D_ through proposing a method that doesn’t depend on Navier governing equation, then developed new algorithms to estimate YS and UTS for many pure metals and alloys. Also, the proposed method provides another advantage which the use of a single probe to generate and receive C_L_, while the other studies, which use E_D_ equation^16–21,23^, need two types of probes one for generating and receiving C_L_ and the other is for generating and receiving shear velocity (C_S_), where ν in equation E_D_ is, $\left(v=\frac{{C}_{L}^{2}-2{C}_{s}^{2}}{2({C}_{L}^{2}-{C}_{s}^{2})}\right)$. And this means that the proposed technique could efficiently reduce the random error, cost, besides the main goals which are the calculation of YS and UTS and drawing the stress–strain curve by using acoustic wave properties.

This study proposed four steps to achieve targeted goals. In the first one, the pure metals and alloys specimens were classified according to their crystal structure (BCC, FCC, and Hex). Secondly, Tr was calculated by measuring C_L_ and ρ. Then, a polynomial relation between the Tr and each of E, YS, and UTS (for each crystal structure type) was individually constructed. In the final step, E, YS, and UTS were calculated and graphed using the programmed algorithms.

## Methods

### Analysis model

In the acoustic tests, Tr is an important parameter between any two connected elements. This parameter depends on the acoustic impedances of these connected materials

1 $$Tr=2{Z}_{2}/\left({Z}_{1}+{Z}_{2}\right)$$

where Z_1_ and Z_2_ are the acoustic impedances for any two connected materials. According to Eq. (1), this study selected the magnesium (Mg) to be Z_1_ because Mg has the lowest acoustic impedance among the sold metals ${({Z}_{Mg}=Z}_{1}=9.9761\times {10}^{6}\frac{\text{Kg}}{{\text{m}}^{2}\text{s}} )$, while Z_2_ is the acoustic impedance of any other test specimen $({Z}_{2}={Z}_{sp})$. Therefore Eq. (1) becomes:

2 $$Tr=\frac{\left(39.9{Z}_{SP}\right)}{\left(1.5+{Z}_{SP}\right)\left({Z}_{SP}+9.97612\right)}$$

This study found there is auniform relationship between YS and UTS: with Tr if the metals were classified according to their crystal structure.

**Figure 1:**
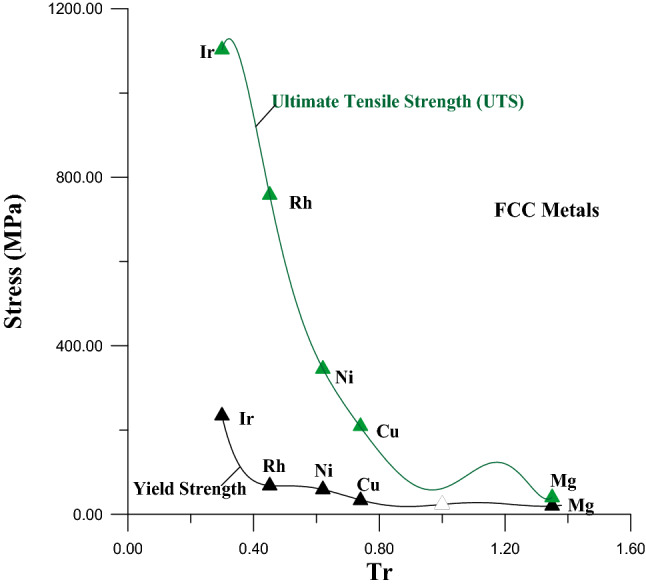
Relationship of UTS and YS with Tr for FCC metals.

In Fig. 1 the values of Tr, of metals that have FCC crystal structure, calculated from Eq. (2), while the values of the YS and UTS for these metals werecollected from references^25–27^. Figure 1 represents the relationship between thesevalues of YS and UTS: with their Tr values for FCC metals.

The Eqs. (3) and (4) were found by using the curve fitting method for the curves in Fig. 1. Equations (3) and (4) indicate there is a disciplined physical relationship between the YS and UTS: with Tr, where the values of YS and UTS decrease with increasing the values of Tr.

3 $$\begin{aligned}{YS}_{FCC} & =4{,}274.76-48701.1\times Tr+24{,}1443\times \left({Tr}^{2}\right)-635{,}316\times \left({Tr}^{3}\right) \\ & \quad +953{,}657\times \left({Tr}^{4}\right)- 818{,}338\times \left({Tr}^{5}\right)+373{,}112\times \left({Tr}^{6}\right)-70{,}012.1\times \left({Tr}^{7}\right)\end{aligned} \dots \dots$$

4 $$\begin{aligned}{UTS}_{FCC}&=40{,}151-419{,}670\times Tr+1{,}840{,}250\times \left({Tr}^{2}\right)-4{,}300{,}940\times \left({Tr}^{3}\right) \\ & \quad +5{,}788{,}240\times \left({Tr}^{4}\right)-4{,}502{,}280\times \left({Tr}^{5}\right)+1{,}881{,}010\times \left({Tr}^{6}\right)-326{,}639\times \left({Tr}^{7}\right)\end{aligned} \dots \dots$$

**Figure 2:**
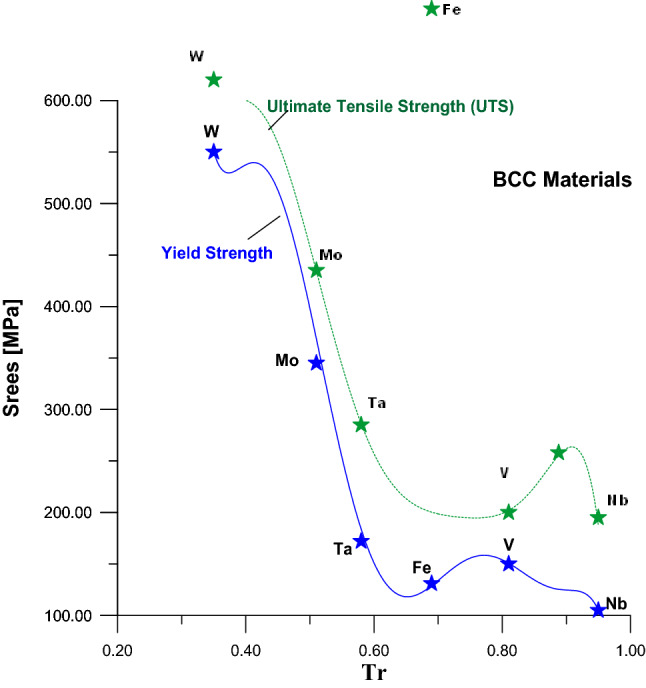
Relationship between UTS and YS with Tr for BCC metal.

Also, the values of Tr of Fig. 2 were calculated by using Eq. (2) and the values of YS and UTS were collected from the same references^25–27^. Figure 2 illustrated the relationships between YS and UTS from the side and their values of Tr from another side for metals that have BCC crystal structure. The Eqs. (5) and (6) represent the mathematical expression of these two relationships shown in Fig. 2

5 $${YS}_{BCC}=127{,}772 -\left(15{,}88710\times Tr\right)+\left(8242590\times {Tr}^{2}\right)-\left(23{,}016{,}300\times {Tr}^{3}\right)+\left(37{,}392{,}900\times {Tr}^{4}\right)-\left(35{,}434{,}900\times {Tr}^{5}\right)+\left( 18{,}188{,}700\times {Tr}^{6}\right)-\left(3{,}912{,}140\times {Tr}^{7}\right)\dots \dots \dots \dots \dots \dots $$

6 $${UTS}_{BCC}=-2{,}336.06+15{,}257.1*Tr-6{,}856.14*\left({Tr}^{2}\right)-71{,}417.2*\left({Tr}^{3}\right)+120{,}448*\left({Tr}^{4}\right)-55{,}118.3*\left({Tr}^{5}\right)\dots \dots \dots .$$

**Figure 3:**
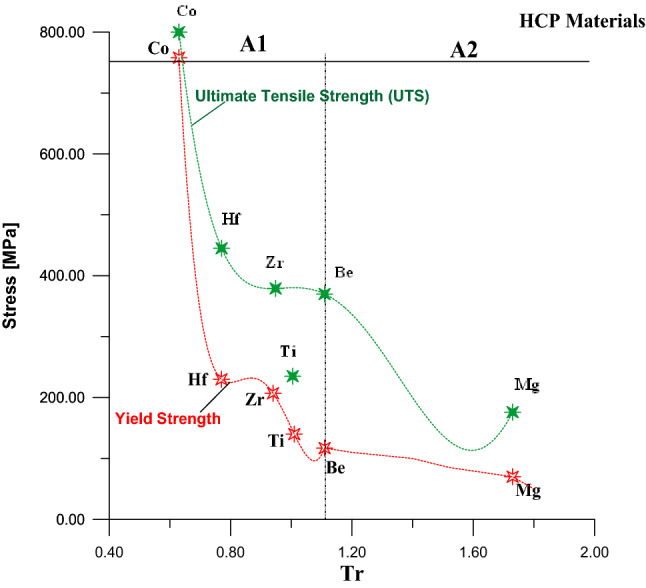
Relationship between yield stress and Tr for HCP metal.

Figure 3 was divided into two parts A1 and A2 to find the equivalent relationship between Tr and the YS for HCP metals. In the same figure and same context, there is a uniform relationship between UTS and Tr for HCP metals. Equation (7a) represents the part A1in the Fig. 3 for metals that have values of Tr ≤ 1.11, while Eq. (7b) represents the part A2 for metals that have values of Tr > 1.11.

For section A1:

7a $${YS(A1)}_{HCP}=87{,}005.7-38{,}8401\times Tr+ 647{,}564\times {Tr}^{2}-476{,}290\times {Tr}^{3}+ 130{,}272\times {Tr}^{4}\dots \dots \dots .$$

Section A2:

7b $${YS\left(A2\right)}_{HCP}=8{,}658.89-25{,}203.4\times \left(Tr\right)+ 27{,}714.7\times \left({Tr}^{2}\right)-13{,}444.7\times \left({Tr}^{3}\right)+2{,}420.17\times \left({Tr}^{4}\right)\dots \dots .$$

Equation (8) indicates the relationship between UTS and Tr, as shown in green color in Fig. 3, for HCP metals:

8 $${UTS}_{HCP}=20576-81541.2*\left(Tr\right)+127710*\left({Tr}^{2}\right)-96409.6*\left({Tr}^{3}\right)+34862.6*\left({Tr}^{4}\right)-4817.61*\left({Tr}^{5}\right)$$

The collected data in this section (Table 1) were gathered from authorized sources^25,28,29^. The Eq. (9) calculates the convergence (Conv1) between the values of the standard YS coming from tensile tests, according to ASTM (YS_ASTM_), and the YS values calculated from the proposed method (YS_cal_) in this study (Eqs. 3, 5, 7).

**Table 1:** Mechanical properties classification of pure metals according to their crystal structure ^25–27^.

Crystal structure	Metal name	*C*_*L*_[ASTM]	*ρ* (kg/m^3^) [ASTM]	*Z* (kg/m^2^ s) *10^6^	Tr	*YS*_*ASTM*_*, *MPa	*YS*_*calc*_*, *MPa	*Conv1.*%	*UTS*_*ASTM*_*, *MPa	*UTS*_*cal*_*, *MPa	*Conv2.*%	E_cal,_ GPa	*Conv3.*%
FCC	AL	6,320	2,710	17.127	1.3535	12	14.014	83.215	45	48.787	91.582	68.136	90.1
	Ge	5,450	5,470	29.811	0.9547	130	130.62	99.518	150	158.39	94.403	163.13	83.47
	Thorium	2,850	11,720	33.402	0.8803	144	144.25	99.825	217	203.83	93.93	57.597	80
	Cu (C10200) Cu(C10100)	4,660	8,941	41.665	0.7457	145	144.5	99.655	280	285.54	98.018	136.85	94.72
	NI	5,515	8,890	49.028	0.6561	150	144.77	90.057	400	407.54	98.113	218.78	90.05
	Rh	6,190	12,410	76.817	0.4509	200	199.91	99.958	700	699.9	99.986	371.98	98.14
	Ir	5,380	22,650	121.85	0.2989	234	233.96	99.986	1,000	999.67	99.967	443.62	84.01
BCC	Nb	3,480	8,570	29.82	0.95	105	101.62	96.667	195	183.09	93.894	103.98	99.036
	V	6,000	6,160	36.96	0.81	150	158.06	94.621	200	207.09	96.450	132.36	99.951
	Fe	5,900	7,800	46.02	0.69	131	141.29	92.138	689	147.33	21.383	212.51	99.991
	Ta	3,400	16,654	56.62	0.58	172	178.32	96.325	285	277.95	97.527	173.76	99.960
	Mo	6,370	10,220	65.1	0.51	345	322.79	93.56	435	414.10	95.196	339.67	99.979
	W	5,180	19,300	99.97	0.35	550	550.51	99.906	620	624.59	99.258	407.43	99.991
HCP	Mg	5,740	1,738	9.9761	1.7383	69	70.952	97.17	176	184.22	95.326	41.681	92.625
	Be	12,800	1,850	23.68	1.1148	117	124.44	93.641	370	368.74	99.660	278.61	97.078
	Ti	6,100	4,450	27.145	1.0185	140	131.51	93.942	235	380.2	38.209	122.68	97.759
	Zr	4,650	6,480	30.132	0.9476	207	201.38	97.287	379	378.93	99.984	131.21	64.726
	Hf	3,000	13,310	39.93	0.7705	230	229.85	99.936	445	444.97	99.994	79.49	56.369
	Colt	5,730	8,900	50.997	0.6356	758	707	93.272	800	775.78	96.973	200.13	94.849

9 $$Conv1=100-\sqrt{{[(({YS}_{ASTM}-{YS}_{cal})/{YS}_{ASTM})\times 100]}^{2}}$$

Equation (10) indicates the convergence (Conv2) between the standard ultimate tensile strength from ASTM (UTS_ASTM_) and the ultimate tensile strength calculated from the proposed method (UTS_cal._)

10 $$Conv2=100-\sqrt{{[(({UTS}_{ASTM}-{UTS}_{cal})/{UTS}_{ASTM})\times 100]}^{2}}$$

The convergence ratio between YS_ASTM_ and YS_cal_ is shown in column Conv1% in the Table 1, while the convergence between UTS_ASTM_ and UTS_cal_ is as shown in a column Conv2% in Table 1. The last two columns in Table 1 calculated from Eq. (8) in the references^22,24^.

The equations from (3–8) were programmed as shown in the index (A), where this program has three input values (C_L_, ρ, and the type of crystal structure (Cy)). The values in the red color in Table 1 represented the faults of this program (in index A).

**Figure 4:**
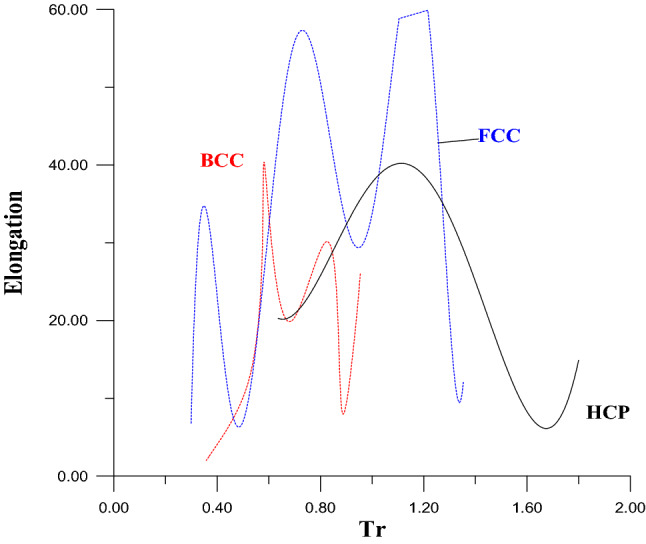
The relations between values of elongations and Trs for pure metals.

To compare the results of this program with the other works, and to show the program's ability to draw a stress–strain curve not only for pure metals but also for alloys that have purity more than 99.95% therefore, more information should be added. This information is the elongation of these metals. Figure 4 shows the undisciplined relationships between elongation values with Tr contrary to YS and UTS which have disciplined relations with Tr. It is worthily mentioned, that Tr values, in Fig. 4, calculated from Eq. (2); while the elongation values were collected from references^25,28,29^.

## Experimental model

The practical methodology of this research depends on three steps:

1. Make sure from crystal structure by using the X-Ray diffraction (XRD) test for each specimen.
2. Calculation Tr for each sample through measurement the C_L_ and ρ for each sample, then, calculate YS_cal_ and UTS_cal_ for these samples by using the Eqs. (3–8).
3. Comparison of the results coming from step 2 with the standard values of YS and UTS for each sample (Mg, Ni, and Nb) according to ASTM.

In this research, the three specimens were selected to prove the theoretical part. Mg specimen was as a sample for HCP crystal structure. And, Ni specimen was selected as a sample of FCC crystal structure; while Nb specimen was as a sample for BCC crystal structure (Supplementary Information 1).

**Figure 5:**
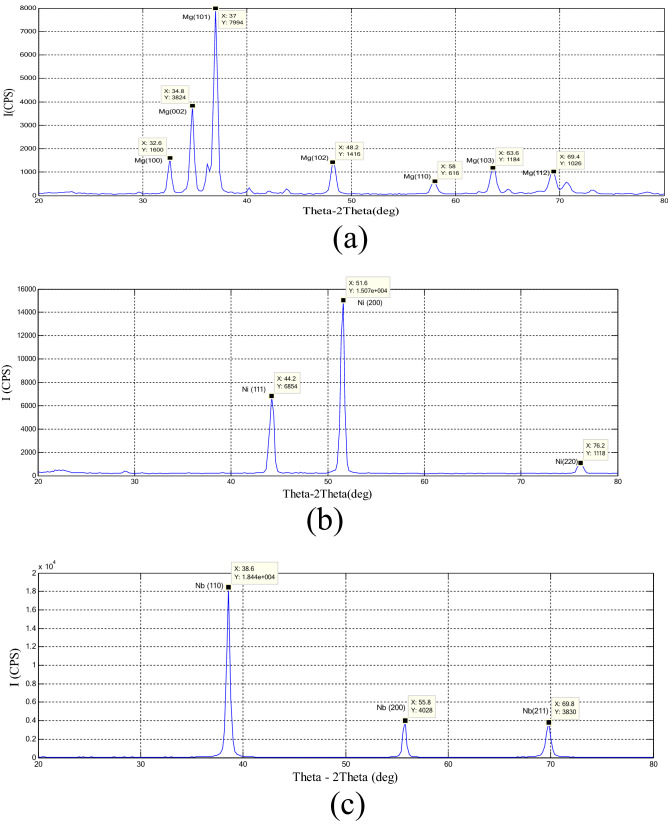
(**a**) XRD for Mg specimen, (**b**) XRD for Ni specimen and (**c**) XRD for Nb specimen.
Step-2: Measuring C_L_ and ρ then calculation Tr
  In this research, the echo pulse technique was used to measure the time of flight of the wave for the same specimens mentioned in step-1. This time is calculated from Eq. (11)^30,31^
  11 $${t}_{TOF}=\frac{2L}{{C}_{L}}+2{t}_{o}$$
  12 $$\therefore {C}_{L}=\frac{2L}{({t}_{TOF}-2{t}_{o})}$$
  where t_TOF_ is the time of flight of the wave in the specimen, L is the specimen length, and t_o_ is the wedge delay of the used probe^32,33^. The t_o_ for the used probe in this research equals 9µsec.This value (9µsec) was calibrated by using the digital ultrasonic thickness tester GM100 where the natural frequency of this probe equals 2.5 MHz.

Step-1: XRD test
  Small samples of Mg, Ni, and Nb were prepared to be suitable for XRD 6,000 SHIMDZU device for doing the XRD test. The results of these tests, as shown in Fig. 5, proved the purity of these samples and proved identical to the crystal structure of these specimens with the crystal structure of these specimens in Table 1.

The Ultrasonic Pulse UP200 (OSUN) is used to generate the electric pulses at a frequency equal to 1,000 Hz, the duration of the mode is 0.1 and the output voltage equals to 200 V in to excite the probe to generate ultrasonic waves. The oscilloscope type of DSEX1102A (100 MHz) oscilloscope was used in this study. Before starting, an ultrasonic gel is placed to get a good connection between the probe and sample to maintain signal strength.

It is worth mentioning, that this oscilloscope has a high sampling rate (2G samples/sec). This advantage gives the system the ability to detect the echo signal for the specimen with thickness 1 mm and this advantage gives preference over the device 38DL PLUS, which cannot detect the thickening less than 4 mm^23^. Also, this oscilloscope gives directly the delay in time between the electric excitation signal and the returned signal (the returned signal from the test specimen) and this is another feature for this oscilloscope.

**Figure 6:**
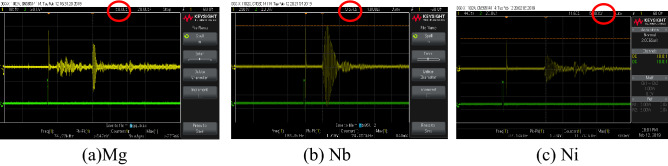
The ultrasonic test for Mg, Nb, and Ni.

Before putting the probe on the specimen, the ultrasonic gel was put on the surface of this specimen to avoid the effect of the air blanks between the probe and the specimen and to guarantee good contact between them. After putting the probe on the specimen, directly the t_TOF_ appears in the middle of the oscilloscope screen as shown in Fig. 6. The red circle in this figure shows the value of t_TOF_ equals 50 µs for the Mg specimen while the values of t_TOF_ are 9.57 µs and 11.6 µs for Nb and Ni respectively as show.

It is worth mentioning the beginning of calculation of the t_TOF_ is from the moment of receiving the echo signal as shown in all Fig. 5. Table 2 involved the details and description of the used specimens in this study. Also, the values of Tr for all these specimens were calculated in this table too. Also, this table refers to the equations that calculate the values of YS and UTS.

**Table 2:** Tr, YS, and UTS calculations.

Metal name	Dimensions of specimens Volume (V) = L*W*D, m^3^	Mass (m), kg	Density (ρ) $\rho =m/V$	The measured time of flight (t), s	Tr calculations	YS_calculated_ (MPa)	UTS_calculated_ (MPa)
Ni (FCC)	$0.05\times 0.0495\times 0.0075=1.8.56\times {10}^{-6}$	0.16502	8,890	$\left(11.6-9\right)\times {10}^{-6}=2.6\times {10}^{-6}$	${C}_{L}=\frac{L\times 2}{t}=\frac{0.0075\times 2}{2.6\times {10}^{-6}}$ ${C}_{L} =5769$ $Z={C}_{L}\times \rho =51.38\times {10}^{6}$ In Eq. (2) $Tr=\frac{\left(39.9\times 51.38\right)}{\left(1.5+51.38\right)\left(10.07+51.381\right)}$ $Tr=0.631$	147.8 (in Eq. (3) because it is FCC)	451.17 (in Eq. (4) because it is FCC)
Nb (BCC)	$0.02\times 0.02\times 0.001=0.4\times {10}^{-6}$	0.00342	8,570	$\left(9.57-9\right)\times {10}^{-6}=0.57\times {10}^{-6}$	${C}_{L}=\frac{L\times 2}{t}=\frac{0.01\times 2}{0.57\times {10}^{-6}}$ ${C}_{L} =3508$ $Z={C}_{L}\times \rho =30.06\times {10}^{6}$ In Eq. (2) $Tr=\frac{\left(39.9\times 30.06\right)}{\left(1.5+30.06\right)\left(47.8+30.06\right)}$ $Tr=0.9491$	109 (in Eq. (5) because it is BCC)	197.4 (in Eq. (6) because it is BCC)
Mg (HCP)	$0.1185\times 0.0915\times 0.0365$=3.9576 $\times {10}^{-4}$	0.69	1,743.47	$\left(50-9\right)\times {10}^{-6}=41\times {10}^{-6}$	${C}_{L}=\frac{L\times 2}{t}=\frac{0.1185\times 2}{41\times {10}^{-6}}$ ${C}_{L} =5780$ $Z={C}_{L}\times \rho =10.07\times {10}^{6}$ In Eq. (2) $Tr= \frac{\left(39.9\times 10.07\right)}{\left(1.5+10.07\right)\left(10.07+9.9761\right)}$ $Tr=1.7319$	70.22 (in Eq. (7) because it is HCP and $Tr\ge 1.11$)	177.66 (in Eq. (8) because it is HCP

## Results and discussion

**Figure 7:**
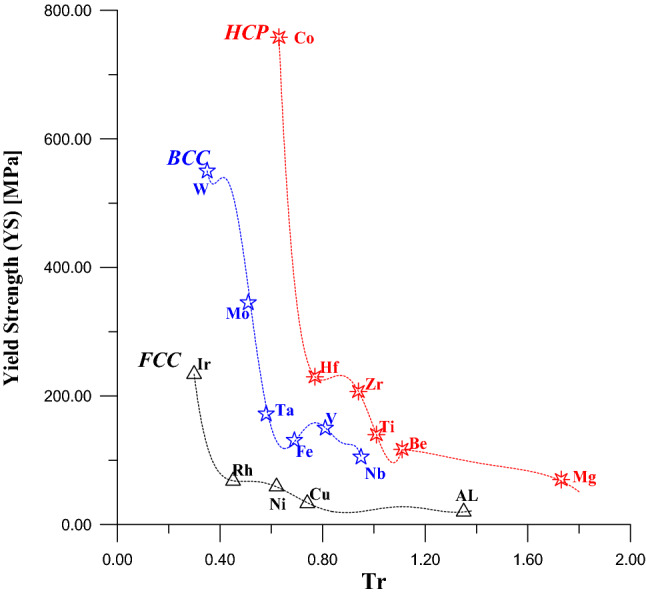
A comparison in values of yield strength among FCC, BCC, and HCP.

**Figure 8:**
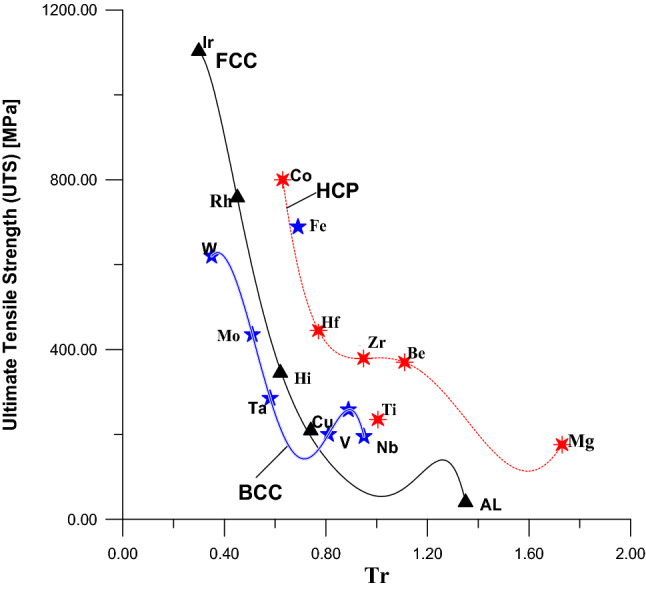
A comparison in values of ultimate tensile strength among FCC, BCC, and HCP.

Figures 7 and 8 indicate the comparison in values of the yield strength and ultimate tensile strength with Tr for the three types of crystal structures (FCC, BCC, and HCP) respectively. It is so clear that the metals that have HCP crystal structure are strongest among the metals that have FCC and BCC crystal structures for the same values of Tr.

The previous studies proved that the crystal structure of iron (Cry-Stru-Fe) has different behavior depending on the environments surrounding him. At room temperature the Cry-Stru-Fe is BCC, but when the temperature is rising until reaches 1,200 °C, the Cry-Stru-Fe changes from BCC to FCC. In the same context^34,35^, had succeeded in changing the Cry-Stru-Fe to HCP at a pressure equals to 360 GPa. On the other hand, the pure titanium crystal structure (Cry-Stru-Ti) exists in two crystallographic forms, first, one as HCP at the room temperature, and BCC at 883 °C and this what is known as beta (β) phase^36^.

This study found these two behaviors (phase transition of crystal structure) for Fe and Ti exists not only at increasing the pressure and temperature, however, these behaviors also exist in the ultimate tensile point. The comparison between the Figs. 7 and 8 illustrates this behavior of Cry-Stru-Fein the tensile testing, where the Cry-Stru-Fe is BCC at the yield point, as shown in Fig. 7, however, when the applied load is increased until reaches to the ultimate load point the Cry-Stru-Fe become so close to HCP group as shown in Fig. 8. In the same context and same comparison, the pure titanium at yield point Cry-Stru-Ti, in Fig. 7, is HCP, however, it becomes so close to BCC when the applied load is increased to the ultimate load point as shown in Fig. 8. And this is one of the new findings of this study about the behavior of Fe and Ti.

The tensile tests are energy added to the sample, this energy compels the crystal structure to distortion and if the metal has the ability on phase transition such as Fe and Ti, this may be caused by changing the crystal structure of Fe from BCC to HCP and Ti from HCP to BCC.

**Figure 9:**
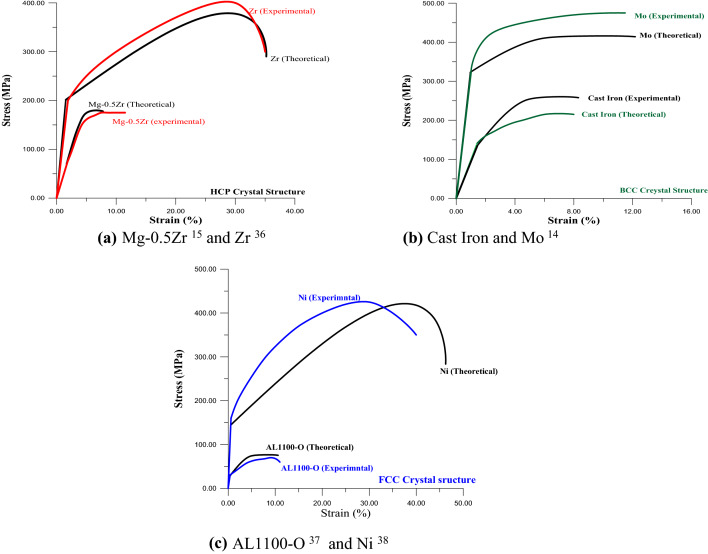
comparison between results coming from the proposed program and other papers^20,21,37–39^.

Figure 9a,b shows a good match in the behavior of curves coming from the proposed program and experimental stress–strain curves of the previous studies. Also, there is an excellent match in stress and strain curves values (E, YS, UTS, and elongation) for curves included in Fig. 9a,b. This match in values of E, YS, UTS, and the elongation is within range of (88 ~ 95)%.

Figure 9c includes two curves first one belongs to the stress–strain curve for Ni. Even though the match in values of E, YS, and UTS for this curve (Ni curve) is quite good, as shown, however, the divergence of these two curves from yield point to ultimate point is around 30%. This divergence of this area of the curve belongs to the mathematical approximation that was used to draw the plastic deformation as shown in equations below:

$$ {\text{EE}} = {\text{YS}}/\left( {\text{E}} \right); $$

$$ {\text{th }} = {\text{ linspace}}\left( {\left( {{2}*{\text{pi}}} \right)/{2},{\text{ pi}}/{6},{ 1}0} \right); $$

$$ {\text{bet}} = {\text{ linspace}}\left( {\left( {{\text{pi}}/{2}} \right), \, 0,{ 1}0} \right); $$

$$ {\text{R }} = {\text{ UTS}} - {\text{YS}}; $$

$$ {\text{dd}} = {\text{Elongation}} - {\text{EE}}; $$

$$ {\text{x1 }} = {\text{ dd}}*{\cos}\left( {{\text{bet}}} \right) + {\text{EE}}; $$

$$ {\text{y1 }} = {\text{ R}}*{\sin}\left( {{\text{th}}} \right) \, + {\text{ YS}}; $$

These sets of equations, which included in the program in Index (A), are constant for all ductile metals. This approximation does not affect the significant values in the stress–strain curve such as E, YS, and UTS, therefore it was regarded as an acceptable result. In the same figure, the match of the stress–strain curve of AL1100-O is around 90%. The 10% difference of stress–strain curve may happen from using different types of instruments or random error^40^.

The stress–strain curve can be estimated by knowing specific points in a stress–strain curve such as (0,0) and point of the elastic limit (ɛ = YS/E, YS), UTS, and the elongation. As was mentioned before, three variables must be entered into the program; these are C_L_, ρ, and type of crystal structure (Cy) to estimate the stress–strain curve. To check the ability and accuracy of this program, the stress–strain curves, for three high purity alloys, published in other papers^20,21,38^ were selected to show the identical among of those curves and the curves producing from this program. It worth mention, these alloys (AL1100-O, Gray cast Iron and Mg-0.5Zr) do not exist in Table 1. Table 3 includes C_L_, ρ, and type of crystal structure for these three alloys. Also, this table includes three alloys that were not drawn however; they match with ASTM in E, YS, and UTS. Figure 9 illustrated these curves and the matching among them. Also, three stress–strain curves for the metals (Ni, Mo, and Zr), from papers^20,37,39^already exist in Table 1, were drawn in this figure too. Anyone can check the ability of this program for the alloys: Ni 233, Ni200, AL1199-O, and AL2014-O by using the data of them (C_L_, ρ, and Cy) and draws the stress–strain curves for these alloys.

**Table 3:** Properties of alloys unlisted in Table 1.

Alloys (99.95%)	*C*_*L*_(m/s) [ASTM]^41^	*ρ* (kg/m^3^) [ASTM]^41^	Crystal structure	Figure
Mg-0.5Zr	5,790	1,740	HCP	9-a
Cast Iron	4,600	7,200	BCC	9-b
AL1100-O	6,350	2,710	FCC	9-c
Ni 233	5,515	8,890	FCC	Did not sketched
Ni 200	5,810	8,890	FCC	
AL1199-O	6,320	2,710	FCC	
AL(2014-O)	6,310	2,800	FCC	

It is worth mentioning that some studies have pointed out a relation between the plastic deformation in the tensile test, the crystal structure, the grain size, and the surface energy of the material, especially in nanomaterials^42–45^. In the same context, Lu et al.^46^ linked between these properties and attenuation of the acoustic waves. The view of ^46^ and view of this study, refer to the possibility of using the acoustic wave properties especially the attenuation in nanostructure materials field and its applications. This study has put the advance step for studying the changing of acoustic impedance with changing crystal structures. And this will open the door for future studies to use the acoustic impedance to study the relation among grain size, grain boundary types, toughness, YS and UTS for a model of the alloys with complex phases or complex compositions.

## Conclusion

The evaluation of YS and UTS by using Tr of the acoustic wave is important for the companies that make an order for purchase the long or big size specimens, such as pipeline, where those big specimens couldn't be tested directly by using normal tensile tests, until doing many of mechanical works. A novel algorithm for predicting mechanical properties of pure metals and alloys has been developed in this study. It depended on three experimental parameters, which are longitudinal wave velocity of metal, density, and crystal structure. The algorithms were also programmed in a MATLAB code to predict metals’ stress–strain relation. Results revealed that for metals that are grouped according to their crystal structure, a disciplined relation between their Trs and mechanical properties could be found. Also, the results proved the excellence of the model is with 85–99% prediction accuracy of ASTM. Furthermore, acoustic impedance and crystal structure were found to have a vital rule in estimating mechanical properties such as YS, UTS, and E. These properties were found to vary linearly with the acoustic impedance and inversely with the pressure transmission coefficient. Further, results showed that metals with HCP crystal structure had higher mechanical properties even for the same acoustic impedance. Moreover, the study reported a phase transition in some of the metals’ crystal structures when loaded up to ultimate tensile stress. For steel, the phase transition was from BCC to FCC, while in titanium the variation was HCB to BCC.

## Supplementary information

Supplementary Information.
