# Supplementary material for: Estimation of the ultimate tensile strength and yield strength for the pure metals and alloys by using the acoustic wave properties
Source: Sci Rep. 2020 Jul 29;10:12676. doi: 10.1038/s41598-020-69387-z (PMC7391784; doi:10.1038/s41598-020-69387-z)
Supplement: Supplementary file 1 — Supplementary Information. [file 41598_2020_69387_MOESM1_ESM.docx]

# Estimation of the Ultimate Tensile Strength and Yield Strength

For the Pure Metals and Alloys by Using the Acoustic Wave Properties

Arshed Abdulhamed Mohammed^1^, Sallehuddin Mohamed Haris^2^Wessam Al Azzawi^1^

Department of Materials Engineering College of Engineering University of Diyala^1^

Baquba, Diyala- Iraq

Department of Mechanical & Manufacturing Engineering, Faculty of Engineering & the Built Environment- University Kebangsaan Malaysia^2^

43600 UKM Bangi, Selangor, Malaysia

Email: (Arshed)[arshad_ald@yahoo.com](mailto:arshad_ald@yahoo.com)Tel: +9647712006187; Fax: +60389259659

**Appendix**

**The Program**

**clc**

**%If the crystal structure (Cy) of materials is FCC then Cy=1. Cy=2 if BCC and % Cy=3 if HCPFCC=1, BCC=2 and HCP=3**

**Cy=3; % Input**

**P=1740; % Input**

**CL =5790; % Input**

**Z= (P*CL)/10^6**

**Tr=(39.9*Z)/((1.5+Z)*(Z+9.97612))**

**if Z>=50.99**

**E=((-5.66211+73.0387*Tr-275.856*(Tr^2)+425.253*(Tr^3)-234.885*(Tr^4))/P)*(10^7)**

**elseif Z>29 && Z<50.99**

**E=((-6.86293+58.1587*(Tr)-102.833*((Tr)^2) +52.8154* ((Tr)^3))*10^6)/P**

**elseif Z<= 29**

**E =(125330000-460076000*(Tr)+ 675849000*(Tr^2)-493364000*(Tr^3)+178397000*(Tr^4)-25525800*(Tr^5))/P**

**End**

**sprintf('%.2f',Tr)**

**if Cy == 1**

**YS=4274.76-48701.1*Tr+241443*(Tr^2)-635316*(Tr^3) +953657*(Tr^4)-818338*(Tr^5)+373112*(Tr^6)-70012.1*(Tr^7)**

**UTS=40151-419670*Tr+1840250*(Tr^2)-4300940*(Tr^3) +5788240*(Tr^4)-4502280*(Tr^5)+1881010*(Tr^6)-326639*(Tr^7)**

**Elong=-9733.76+ 107627*(Tr)-482622*(Tr^2)+1141490*(Tr^3)-1542520*(Tr^4)+1195950*(Tr^5)-494744*(Tr^6)+84585.9*(Tr^7)**

**elseif Cy == 2**

**YS=127772 -(1588710*Tr)+8242590*(Tr^2 )- 23016300*(Tr^3) +37392900*(Tr^4) -35434900*(Tr^5)+18188700*(Tr^6)-3912140*(Tr^7)**

**UTS=-2336.06+15257.1*Tr-6856.14*(Tr^2)-71417.2*(Tr^3) +120448*(Tr^4)-55118.3*(Tr^5)**

**Elong= 82696.5 -807505*(Tr)+3198520*(Tr^2)-6599990*(Tr^3)+ 7503080*(Tr^4)-4465200*(Tr^5)+1088620*(Tr^6)**

**elseif Cy == 3**

**UTS=20576-81541.2*(Tr)+127710*(Tr^2)-96409.6*(Tr^3) +34862.6*(Tr^4)-4817.61*(Tr^5)**

**Elong=463.981-1906.27*(Tr)+2883.43*(Tr^2)-1794.42*(Tr^3)+ 391.038*(Tr^4)**

**%for section A1**

**If Tr<=1.11**

**YS=87005.7-388401*Tr+ 647564*(Tr^2)-476290*(Tr^3)+ 130272*(Tr^4)**

**Elseif Tr>1.11**

**%For Section A2:**

**YS=8658.89-25203.4*(Tr)+ 27714.7*(Tr^2)-13444.7*(Tr^3) +2420.17*(Tr^4)**

**end**

**end**

**if Elong> 15**

**EE=YS/(E);**

**th = linspace((2*pi)/2, pi/6, 10);**

**bet= linspace((pi/2), 0, 10);**

**R = UTS-YS;**

**dd=Elong-EE;**

**x1 = dd*cos(bet)+EE;**

**y1 = R*sin(th) + YS;**

**yy=[0 YS y1 ];**

**xx=[0 EE x1 ];**

**figure (1)**

**plot (xx,yy)**

**xlabel('Strain (%)'), ylabel('Stress (MPa)');**

**else**

**EE=YS/(E)**

**th = linspace((2*pi)/2, pi/2, 6)**

**bet= linspace((pi/2), 0, 100);**

**dd=Elong-EE**

**R = (UTS-YS);**

**y1 = R*sin(th) + YS**

**OO=Elong/2**

**TT=OO/5**

**s=EE**

**for k=1:6**

**s=s;**

**x1(k)=s**

**s=s+TT**

**end**

**yyy=[0 YS y1 UTS];**

**xxx=[0 EE x1 Elong];**

**figure (2)**

**plot (xxx,yyy)**

**xlabel('Strain (%)'), ylabel('Stress (MPa)');**

**end**
